# Determining the distance from the lingual frenum anterior attachment to the lower incisors’ incisal edges

**DOI:** 10.15171/joddd.2016.041

**Published:** 2016-12-21

**Authors:** Farhang Mahboub, Soodabeh Kimyai, Elahe Molavi

**Affiliations:** ^1^Dental and Periodontal Research Center, Tabriz University of Medical Sciences, Tabriz, Iran; ^2^Assistant Professor, Department of Prosthodontics, Faculty of Dentistry, Tabriz University of Medical Sciences, Tabriz, Iran; ^3^Professor, Department of Operative and Esthetic Dentistry, Faculty of Dentistry, Tabriz University of Medical Sciences, Tabriz, Iran; ^4^Postgraduate Student, Department of Pediatric Dentistry, Faculty of Dentistry, Tabriz University of Medical Sciences, Tabriz, Iran

**Keywords:** Lingual frenum, mandibular incisors, vertical dimensions

## Abstract

***Background.*** Occlusal rims are used to determine the jaw relationships in the transverse and vertical dimensions and estimate the inter-occlusal distance in edentulous patients. It is important to find ways to determine the height and shape of the occlusal rims correctly. This study was undertaken to determine the exact distance from the oral cavity floor to the incisal edges of mandibular incisors to serve as a guide for adjusting the height of the mandibular occlusal rim.

***Methods.*** Forty patients were selected and special trays were fabricated to prepare accurate stone casts on which the measurements were made at 0.01-mm accuracy. Two marks were placed on the casts at the incisal edges of mandibular incisors and at anterior attachment of lingual frenum. Then the distance between these two marks was determined on the vertical spindle of a surveyor using a digital Vernier measuring tool and recorded.

***Results.*** The results showed that the mean and standard deviation of the distances between the oral cavity floor and the incisal edges of lower incisors were 14.35 ± 1.68 mm, with a range of 10.2‒17.02 mm. The mean distances in males and females were 15.42 ± 0.97 and 13.28 ± 1.57 mm, respectively. T-test showed significant differences in this distance between males and females, with greater distances in males.

***Conclusion.*** The distance between the oral cavity floor and the incisal edges of mandibular incisors at anterior attachment of lingual frenum might be a proper criterion for the initial adjustment of occlusal rims.

## Introduction

Accurate determination and registration of vertical dimension of occlusion (VDO) is an important step in the treatment of edentulous patients and is considered a very sensitive issue. Vertical relationship of the jaws is determined and registered under a specific condition of the maxilla and mandible. The vertical relationship of the jaws is divided into the vertical dimension of occlusion and the rest position. The vertical dimension of occlusion is established by natural teeth in dentate individuals when they are in occlusion; however, in edentulous patients, such a relationship is maintained by the vertical dimension of dentures while the artificial teeth are in contact with each other. Therefore, the vertical dimension of occlusion should be adjusted while the artificial teeth are in proper contact with each other in edentulous patients.^1^

Various studies have shown, based on evidence, that tooth eruption is associated with tooth attrition. Despite severe attrition of teeth the vertical dimension of occlusion does not decrease due to an increase in the height of the alveolar process.^2^

The techniques used to determine the vertical maxillo-mandibular dimension are divided into two broad categories: The first group consists of mechanical methods, including the use of records before tooth extraction, patient’s lateral radiographs, the stone casts in occlusion, facial measurements, ridge relationships and measurements made on the patients existing prosthetic appliances. The second group consists of physiologic methods, including the physiologic rest position, deglutition, production of sounds and pronunciation of words, the tactile sensation and patient comfort, which are all used to determine the facial height at which the occlusion should be constructed.

All the decisions in relation to the vertical dimension of occlusion should be considered provisional until the teeth are placed on the test occlusal rims. When the teeth/dentures are tested in the oral cavity the way the sounds are produced and the words are pronounced and observation of the facial appearance determine whether the VDO determined by mechanical or physiologic tools is correct or not.^1^

In complete removable dentures, the VDO is an important factor in patients’ satisfaction with their esthetic appearance. In this context, a severely decreased VDO results in patient discomfort and his/her decision not to wear the dentures.^3^Use of stone casts in occlusion, too, is a simple technique to register the vertical overlap relationship and the size and form of the teeth on diagnostic casts mounted on an articulator. These casts demonstrate the amount of space required to align teeth with these sizes in the ridges.^1,4^

In addition, the physiologic rest position is not a very accurate method; however, it will help determine the vertical relationship of the mandible with the maxilla. The interocclusal distance at rest should be 2‒4 mm in the premolar area. The inter-arch space and the rest position can be determined by placing un-removable points or sticky tapes on the patient’s face. If the difference is more than 4 mm, it becomes clear that the VDO is less than that required. If the distance is less than 2 mm, it shows that the VDO is more than it is necessary.^1^ The occlusal rims are adjusted to a point at which the dentist makes sure the inter-arch space is adequate, and the other aspects of favorable vertical dimension (VD) such as patient comfort, esthetic appearance and production of speech (articulator) are met.^1,5^

In the maxilla, the incisive papilla^6^ is an established anatomic landmark that can be used to determine the vertical relation of edentulous patients. Different landmarks have been used to orient the occlusal plane in the mandibular arch, including the retromolar pad, lateral borders of the tongue, the buccinator groove and the commissure of the lips. Unfortunately, they are not stable and reliable anatomic landmarks.^7^ However, the anterior attachment of the lingual frenum can be used for proper positioning of lower anterior teeth in their original position and to establish the level of lower occlusal plane in complete denture patients. In some studies, this distance has been determined in different communities using not very accurate techniques, with different values being reported.^8-10^

The aim of this study was to measure and register the distance between the oral cavity floor and the incisal edges of mandibular incisors at anterior attachment of lingual frenum in an Iranian population at an accuracy rate of 0.01 as a record to adjust the occlusal rims in complete dentures.

## Methods

The study protocol was approved by the Ethics Committee of Tabriz University of Medical Sciences.

Forty patients (20 males and 20 females) referring to the Department of Prosthodontics, Faculty of Dentistry, Tabriz University of Medical Sciences, were selected. The inclusion criteria consisted of the presence of natural mandibular incisors, a correct position of the occlusal plane and absence of tooth attrition and mobility. Patients with Cl II and Cl III occlusal relationships, supra-erupted lower incisors, fractured incisal edges, attrition of the incisal edges or with any extra-coronal restorations in mandibular incisors and patients with ankyloglossia were excluded from the study. To fabricate a partial mandibular denture, after primary impression making by stock tray using alginate (Tropicalgin, Zhermak, Badine Polesine, Italy) and pouring the cast with dental plaster, a spaced special tray was fabricated with the use of self-curing acrylic resin (Acropars Cold Cure Acrylic for Special Tray, Marlic Medical Industries Co, Tehran, Iran). Two layers of modeling wax (Modeling Wax, DENTSPLY Limited, Weybridge, United Kingdom), measuring 2 mm in thickness, were placed in the dentate area and one layer was placed on the edentulous area on the cast to create space. Green compound sticks were used for border molding (Hoffmann Dental Manufaktur GmbH, Berlin, Germany) and the final impression was taken with condensational silicon impression material (Speedex Light Body Polysiloxane, Colten/Whaledent, Switzerland). The impression was poured with Type III stone (Moldstone Dental Stone, Tehran, Iran) with the use of the two-stage technique and the final cast was prepared.

Border molding of the lingual flange of the special tray is important for the selection of the type of the major connector of the partial denture. Therefore, in the resultant cast the depth of the oral cavity floor (the distance between the oral cavity floor at anterior attachment of lingual frenum and the incisal edges of the lower incisors) can be accurately recorded. All the patients were asked to touch their upper lips with the tip of their tongue during border molding and impression procedures. The impression tray was held by the index figure at the site of first molars and second premolars in a bilateral manner during the whole impression taking procedure to prevent the movement of tray during the process. In situations in which there were excessive pressure areas or there was an excessive thickness of the impression material, the impression taking procedure was repeated.^11^

**Figure 1.**
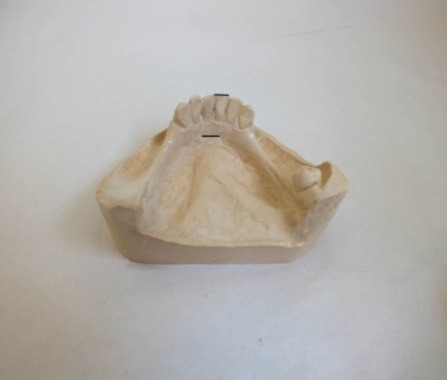
The anterior attachment of lingual frenum at midline and the incisal edge of the lower central incisor were marked on the cast.

The casts were mounted on the platform of a surveyor (Marathon-103, SAEYANG COMPANY, Korea), with the occlusal plane in a horizontal position. To determine the distance from the anterior attachment of lingual frenum to the incisal edges of lower incisors, two marks were placed on the casts. The lowermost mark was placed at anterior attachment of lingual frenum in the midline at the sublingual sulcus and the uppermost mark was placed on the incisal edge of the left or right central incisor (Figure 1).

**Figure 2.**
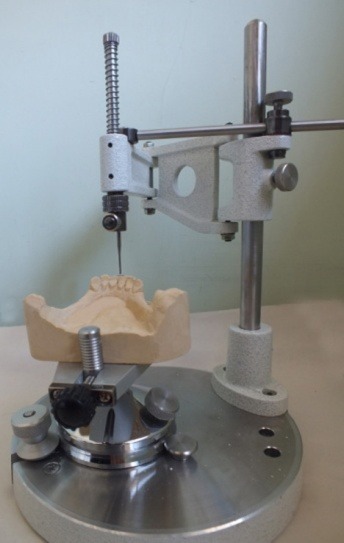
The contact point of the vertical spindle and the cross arm of the surveyor was marked when the analyzer’s spindle touched the lowermost point.

The position of each cast was adjusted to stabilize the metallic plate of the surveyor to contact the teeth at least at three divergent points. The spindle of the surveyor analyzer was lowered so that its tip touched the lowermost point. Then a horizontal mark was placed with the use of a pencil at the contact point of the vertical spindle of the surveyor with the cross arm (Figure 2).

**Figure 3.**
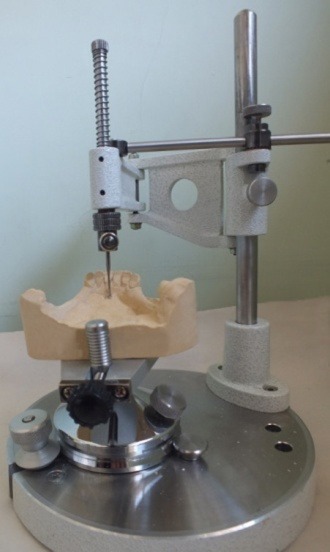
The contact point of the vertical spindle and the cross arm of the surveyor was marked when the analyzer’s tip touched the lowermost point.

Similarly, the second horizontal mark was placed on the vertical spindle of the surveyor where the tip of the surveyor analyzer touched the uppermost mark (Figure 3).

**Figure 4.**
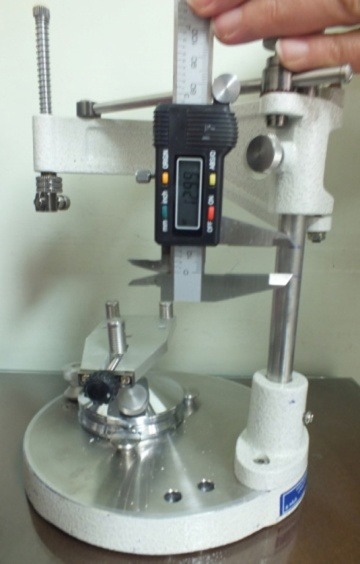
The distance between the two points on the vertical spindle of the surveyor was measured by a digital Vernier device.

The distance between the two marks was measured and recorded with the use of a digital Vernier measuring device accurate to 0.01 mm. The measurements were carried out by a second operator (Figure 4).

The statistical method used was the “mean estimate” technique with SPSS 13. The means, medians, modes and standard deviations were calculated.

Independent samples t-test was used to evaluate the relationship between gender and the distance between anterior attachment of lingual frenum (AALF) and the incisal edge of lower incisors because the independent variable (input) had two levels and the dependent variable (output) was semi-quantitative. Kappa agreement coefficient was used for independent measurements.

## Results

Pearson’s correlation coefficient exhibited a strong and significant relationship between the observations made by the two observers (Pearson’s coefficient = 0.971; P = 0.000).

The results showed a mean distance of 14.35 ± 1.68 mm, with a range of 10.2‒17.02 mm, between the oral cavity floor at anterior attachment of lingual frenum and the incisal edges of lower incisors. The median was 14.5 mm and no value was repeated. The mean distances between the males and females were 15.42 ± 0.97 and 13.28 ± 1.57 mm, respectively. T-test showed significant differences in distances between males and females, with greater distances in males compared to females (Table 1).

**Table 1:** Comparison of the distances between the oral cavity floor at anterior attachment of lingual frenum and the incisal edges of mandibular incisors

**Gender**	**No.**	**Mean**	**SD**	**T-test**		
				**t-value**	**Degree of freedom**	**P-value**
**Male**	20	15.4220	.97793	5.156	38	.001
**Female**	20	13.2890	1.57050			

## Discussion

An important step in the treatment of edentulous patients is the accurate determination and registration of the vertical dimension of occlusion (VDO), which is defined and determined at the position of occlusion. The VDO in dentate patients is defined when the teeth are in occlusion; however, in edentulous patients it is defined when the artificial teeth touch each other. Therefore, the VDO should be adjusted in edentulous patients in a manner so that the artificial teeth will properly contact each other. Therefore, the VDO should be adjusted while the artificial teeth are in proper contact with each other in edentulous patients.^1,12^ Various studies have shown that tooth eruption is concomitant with tooth attrition. However, despite severe attrition of teeth the VDO does not decrease because the height of the alveolar process increases.^2,13^

In a study by Sir and Yoo,^14^ the distance between the subnasal point and the lowermost point on the chin was measured. In that study, 7 points were selected on the sagittal plane in 50 patients (26 patients with both upper and lower dentures and 24 patients with upper complete dentures or lower partial dentures). The results showed that the VDO in patients was 70.03 mm when they were wearing none of the dentures, with 70.88 when they were wearing both dentures. The VDO was 77.83 mm when only the lower denture was in place, with 70.18 when only the upper denture was in place. Therefore, they concluded that the lower denture alone has an important role in VDO. VDO was registered at 68.81 and 72.15 mm in females and males, respectively, in patients with complete or partial dentures. In addition, it was shown that an increase in VDO might be related to the interaction of dental arches; a decrease in VDO might be related to the expansion of dental arches due to the muscle tonicity and the contour of the lips.^15^ The difference in the VDO between males and females was consistent with the results of the present study.

A review study by Stajnic and Smobad^16^evaluated the possibility of using cephalometric analyses to improve the clinical techniques of VDO determination in the treatment of edentulous patients. They finally suggested that different techniques for VDO determination be combined. VDO is important for all the aspects of prosthodontics. Unfortunately, no specific and scientific technique is available to determine VDO.^16,17^ Patients whose VDO has been determined less than their standard values will have various problems during the complete denture fabrication steps. In a study by Mays,^18^ diagnostic prostheses were used to assess the patients’ ability to tolerate increased VDO and it was concluded that an increase in VDO up to 5 mm did not give rise to any specific discomfort during a 2-month period.

Manukata and Kasai^19^ used extraoral jacks connected to intraoral hydraulic jacks to change VDO of complete dentures to find the most comfortable position of the mandible. They used this technique to determine VDO but it is not possible to use this technique routinely in the clinic because it involves the use of specific and complicated tools.

Zarb et al^13^ reported a mean VDO of 22 mm in the canine sulcus area of the upper jaw, with 18 mm in the lower jaw in the new edition of his textbook; however, the previous edition of the textbook has considered a VDO of 20 mm in both jaws.^1^ In a similar study by Bissasu,^10^ the mean distance between the oral cavity floor at the anterior attachment of lingual frenum and the incisal edges of the lower incisors was reported to be 10.26 mm, which is significantly less than that in the present study. Such a discrepancy in the results might be attributed to impression techniques with low accuracy and small sample sizes in the study by Bissasu^10^ in which stock trays and alginate impression material were used. In another study by Parimala and Prithviraj^8^the mean distance was reported 12.3 mm with no difference between males and females. This discrepancy with the results of the present study could be due to the different impression making techniques and ethnic anatomic variations. The results of the current study strongly correlate with the study of Balasubramanian et al,^9^whoused more accurate modified stock tray for impression making and reported 14.50 mm for the mean space between the anterior attachment of lingual frenum and the incisal edges of the lower incisors.

## Conclusion

According to the results of the current study, the distance from the oral cavity floor to the incisal edges of lower incisors at anterior attachment of lingual frenum might be a proper criterion to determine the most appropriate vertical height of occlusion. In general, elevation of the occlusal rims can be considered 15 and 13 mm in males and females, respectively.

## Acknowledgments

The authors thank the Research Vice Chancellor and Dental and Periodontal Research Center of Tabriz University of Medical Sciences.

## Authors’ contributions

The study was designed by FM and SK, and EM carried out the study procedures. The statistical analyses and explanation of data were carried out by FM. SK and EM were responsible for manuscript preparation. FM critically revised the manuscript for intellectual content. All the authors contributed to the final draft, read and approved the final manuscript.

## Funding

This study was supported and funded by Dental and Periodontal Research Center of Tabriz University of Medical Sciences.

## Competing interests

The authors declare no competing interests with regard to the authorship and/or publication of this article.

## Ethics approval

The study protocol was approved by the Ethics Committee of Tabriz University of Medical Sciences.
